# 19.3 APPLYING COGNITIVE ADAPTATION TRAINING IN FINLAND: INTERIM RESULTS OF THE FINNISH CAT IMPLEMENTATION PROJECT

**DOI:** 10.1093/schbul/sby014.077

**Published:** 2018-04-01

**Authors:** Tuukka Mehtala, Satu Viertio, Eila Sailas

**Affiliations:** 1 HUS; 2 National Institute for Health and Welfare; 3 Kellokoski Hospital

## Abstract

**Background:**

In Finland, approximately 50 000 people have a diagnosis of schizophrenia. In practice 6% of them reside permanently in mental hospitals. There is a national target to reduce the number of psychiatric hospital beds. However, as hospitals are closed there is a tendency to place schizophrenia patients in different types of sheltered housing instead of supporting them to live independently in the community. In the Danish OPUS-study 94 patients with first episode schizophrenia were followed and even those who had attended a vigorous rehabilitation program lived about two and a half months in sheltered housing in the fifth year after their diagnosis. Thus, with deinstitutionalization we are building up a poorly monitored system of sheltered housing for schizophrenia patients. This system may increase chronic need for support, is expensive and marginalizes a large section of people from the community. When service users are asked they usually prefer having their own homes.

Cognitive adaptation training (CAT) is a home-based, manual-driven treatment that utilizes environmental supports and compensatory strategies to bypass cognitive deficits and improve target behaviors and functional outcomes in individuals with schizophrenia. Unlike traditional case management, CAT provides environmental supports and compensatory strategies tailored to meet the behavioral style and neurocognitive deficits of each individual patient. CAT has been shown to be effective in improving service users’ ability live independently.

**Methods:**

The study started in 2014. After formal CAT training the program was implemented in the Hyvinkää Hospital and Helsinki University Central Hospital treatment catchment areas (approx. 1 350 000 inhabitants). For the study we selected patients that were in risk of moving to a more supported housing environment due to the presence of cognitive deficits that threatened their ability to live independently. The only exclusion criteria were heavy alcohol and drug abuse and known aggressive behavior. The outcome measurements include both qualitative and quantitative methods: transfer to a different type of housing, need for hospital treatment, psychiatric rating scales, observed measurements and open interviews, and are measured after 4 months after the start of the intervention, at the end of the 9 month intervention and after a 6 months follow-up period.

**Results:**

We report here preliminary interim results for the patients who have completed the study so far. Altogether 48 patients were selected for the intervention, which was found to be well-received with 7 patients dropping out. The mean age was 38.9 year, with 39.3 % women and 60.4 % men. 27.6 % were living independently, 22.9 % with their parents, and 29.6 % living in some form of sheltered housing. Participants had severe to moderately-severe psychiatric symptoms and functional impairment (mean GAF 47.8, mean SOFAS 54.8). Apathetic was the most common behavioral subtype (70.7 %), with disinhibited (14.6 %) and mixed (14.6 %) subtypes following.

**Discussion:**

Cognitive Adaptation Training can be used to help patients in a wide range of living situations and with severe psychiatric symptoms and functional impairment to maintain their ability to live independently.

